# Review of the genus *Plistobunus* Pocock, 1903, with description of a new species from Hainan Island, China (Opiliones, Laniatores, Epedanidae)

**DOI:** 10.3897/zookeys.112.1110

**Published:** 2011-06-24

**Authors:** Wei-Guang Lian, Chao Zhang, Feng Zhang

**Affiliations:** 1 Department of Laboratory Animal Science, Hebei Medical University; Key Lab of Laboratory Animal Science of Hebei Province, Shijiazhuang, 050017, China; 2 College of Life Sciences, Hebei University, Baoding, Hebei 071002, China

**Keywords:** taxonomy, Arachnida, harvestmen, *Plistobunus*, China

## Abstract

The genus *Plistobunus* Pocock, 1903 and its type species *Plistobunus rapax* Pocock, 1903 are redescribed based on the type material deposited in the British Museum of Natural History (BMNH), London. In addition, a new *Plistobunus* species from Hainan Island is described and illustrated of *Plistobunus columnarius* **sp. n.** The new species is diagnosed by having a row of 12 setiferous tubercles on anterior margin of carapace, and the femur of pedipalpus ventrally with 13 setiferous tubercles in male.

## Introduction

The Epedanidae Sørensen, 1886 includes 73 genera and 188 species, and they are endemic to Asia (Kury 1993). According to Pinto-da-Rocha and Giribet (2007) and Kury (2007), no systematic research to this family in the later decades, although four new species are occasionally being proposed (Kury 2008; Zhu and Lian 2006; Lian et al., 2008; Zhang and Zhang 2010).

This family was removed from the Phalangodidae Simon, 1879 by Kury (1993), based mainly on the presence of a well developed immovable sac (which he called ‘follis’) and the absence of complex introverting structures in the penis (Lian et al. 2008). Kury (2003) recognized the following four subfamilies under Epedanidae: Acrobuninae Roewer, 1912; Sarasinicinae Roewer, 1923; Epedaninae Sørensen, 1886 and Dibuninae Roewer, 1912.

The genus *Plistobunus* of Epedaninae was erected by Pocock in 1903 based on a single male, the type species *Plistobunus rapax* Pocock, 1903. The original description provided by Pocock (1903) was brief and the figure was very schematic. Although Roewer (1912) failed to examine the type species *Plistobunus rapax*, he redescribed the genus characters in detail with the helps of Hirst who was an arachnologist in London. Moreover, the description was mentioned again in Roewer’s voluminous book, as well as the only figure which is from Pocock (Roewer 1923). In 1937, Roewer finally examined, redescribed and illustrated the type specimen. So far, there is no further report for this genus and species.

In 2009, we explored Hainan Island, China, and collected some laniatorid harvestmen specimens by sieving the leaf in the forest. Among the collected specimens, we recognized a species new to science, and describe it under the name *Plistobunus columnarius* sp. n. here. Additionally, we loaned the type specimen of *Plistobunus rapax* from the British Museum of Natural History (BMNH), London, examined, redescribed and illustrated it, and revised the generic characters based on the two species above.

## Materials and methods

Taxonomic methods follow outline proposed by Acosta et al. (2007). The type materials of *Plistobunus* was loaned from the British Museum of Natural History, London, British (BMNH) and was preserved in 75% Industrial Methylated Spirit (IMS), also was examined, drawn and measured under a Tech XTL-II stereomicroscope equipped with an Abbe drawing device. Type specimens of the new species were preserved in 75% ethanol (deposited in the Museum of Hebei University, Baoding, China (MHBU)) and were examined and drawn under a Leica M165c stereomicroscope equipped with drawing tube. The male genitalia were placed firstly in hot lactic acid, followed by distilled water to expand those parts for observation (Schwendinger and Martens 2002). The terminology of the setae on penis follow Ubick and Briggs (2004), except for basal setae of glans, which is presented by current authors. All measurements are given in mm.

The following abbreviations are used in the text: BS, basal sac; DP, dorsal plate; DS, dorsal setae; DSL, dorsal stylar lobe; G, glans; GBS, basal setae of glans; LP, lateral plate; LS, lateral setae; S, stylus; SL, stylar lobe; VP, ventral plate; VS, ventral setae; VSL, ventral stylar lobe.

## Taxonomy

### Plistobunus

Pocock, 1903

http://species-id.net/wiki/Plistobunus

Plistobunus Pocock 1903: 447; Roewer 1912: 232; 1923: 207; 1938: 124.

#### Type species:

*Plistobunus rapax* Pocock, 1903, by original designation.

#### Diagnosis.

Medium-sized epedanines (3.03–3.57) with a long median spine on the ocularium. Carapace with a row of 4–6 setiferous tubercles on each side of the frontal margin. Area II with a pair of spines. Area IV with a median spine. Area IV and all free tergites with a transverse row of hair-tipped tubercles. The proximal segment of chelicera elongated and armed above with numerous tubercles, of which distal one enlarged the largest on the dorsal surface. Pedipalpus elongated; femur of male with 9–13 setiferous tubercles ventrally, a longitudinal row of 7–9 setiferous tubercles dorsally, and with two tubercles on medial side distally; patella of male with two setiferous tubercle disto-medially and three setiferous tubercles ectally. Distitarsus of leg I with two segments. Shaft of penis widened distally. DP conspicuous, VP complex. G protrude sideways beyond the distal penis and near the DP. S is surrounded and protected by SL. BS globular, immovable and entire hidden into truncus.

#### Distribution:

China (Hongkong, Hainan).

#### Remarks.

The male genitalia of *Plistobunus rapax* remains unknown, because the penis was lost (see remarks below). According to study of *Plistobunus columnarius* sp. n., we tentatively supplemented the male genital structure to the generic characters.

### Plistobunus rapax

Pocock, 1903

http://species-id.net/wiki/Plistobunus_rapax

Figs 1–6

Plistobunus rapax Pocock 1903: 447, fig. 2; Roewer 1912: 232; 1923: 207, fig. 236; 1938: 124, fig. 43.

#### Type material examined.

Holotype *♂*, in 75% Industrial Methylated Spirit (IMS), labelled as follows: “56. 113, *Plistobunus rapax* Pocock, Hong Kong” (BMNH 56. 113).

#### Redescription.

**Figures 1–6.**
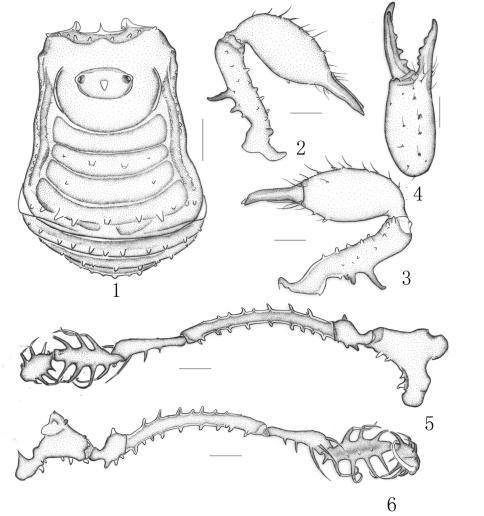
*Plistobunus rapax* Pocock, 1903, male (holotype) **1** Body, dorsal view **2** Left chelicera, medial view **3** Same, ectal view **4** Distal segment of the left chelicera, frontal view **5** Left pedipalpus, ectal view **6** Same, medial view. Scale bars: 0.5 mm (1–6).

Male holotype (habitus see Fig. 1): Coloration. Body yellowish brown and appendages yellow. Lateral margins and free tergites banded with dark brown. Chelicerae dorsally reticulated with dark brown.

##### Dorsum.

Dorsal scutum nearly trapezoid in shape; widest portion at fourth scutal area; anterior margin of carapace armed with a transverse row of four to five setiferous tubercles. Ocularium long oval, armed with a short median spine. Opisthosomal region of scutum with four areas, first area completely smooth, without a median furrow or line; second area has four hair-tipped tubercles, of which two median ones are longer than others; third area covered with two relatively tubercles; fourth area with a transverse row of seven tubercles, of which the median one is longest. Free tergites with hair-tipped granules arraged in a transverse; each lateral margin of the scutum with a longitudinal row of granules.

##### Venter.

Coxae I–III armed with a row of hair-tipped tubercles, additionally coxa I covered with a row of relatively small hair-tipped tubercles. Coxa III with a row of low humps along front and hind margins. Coxa IV with a row of small hair-tipped granules. Some small hair-tipped granules scttered over surfaces of coxae I–IV. Tracheal stigma clearly visible.

##### Chelicera (Figs 2–4).

Proximal segment fairly strong, distinctly armed with two prominent spines dorsally, numerous hair-tipped tubercles scattered over ventral and lateral surface. Second segment distinctly expanded, armed with a row of four strong hair-tipped bifid tubercles on the prodorsal surface. A few hair-tipped granules scattered over the prodorsal surface. Fingers relatively strong, cutting edges dentate (Fig. 4).

##### Pedipalpus (Figs 5–6).

Relatively long and slender. Trochanter with a single setiferous tubercle dorsally, three ventrally. Femur dorsally with a longitudinal row of seven setiferous tubercles; ventrally with a longitudinal row of nine setiferous tubercles; distally with two setiferous tubercles medially. Patella ectally with three setiferous tubercles, disto-medially with two ones. Tibia with three medial and five ectal setiferous tubercles. Tarsus with four setiferous tubercles on both sides of ventral surface. Tibia with a longitudinal row of three granules ventrally. Tarsal claw long, strongly curved.

##### Legs.

All of legs were destroyed and missing but their trochanters ventrally with two hair-tipped tubercles, their femora armed with a row of setiferous tubercles.

##### Penis.

Lost.

##### Measurements.

Body 3.03 long, scutum 2.64 long, 2.25 with at the widest portion; ocularium 0.65 long, 0.35 wide.

#### Distribution.

China: Hong Kong.

#### Remarks.

*Plistobunus rapax* is only known from the type specimen. To make matters worse, the type specimen is in an incomplete state. All legs were missing and the penis is lost. Roewer at first (1912, 1923) did not examine the holotype by himself, and learned from Hirst that all the tarsi of legs in type specimen were missing. According to the description of Pocock, he assumed that the species should be placed in the subfamily Epedaninae, family Phalangodidae. Later Roewer (1937) examined the type specimen, he found that all legs were lost, then he (1938) listed it also in the family Epedanidae, merely based on the original description and Hirst’s information. We can not confirm when the penis was lost, as Roewer did not usually describe and illustrate the structure of penis, because he was unaware of the importance of genital diagnostic characters.

**Figures 7–14.**
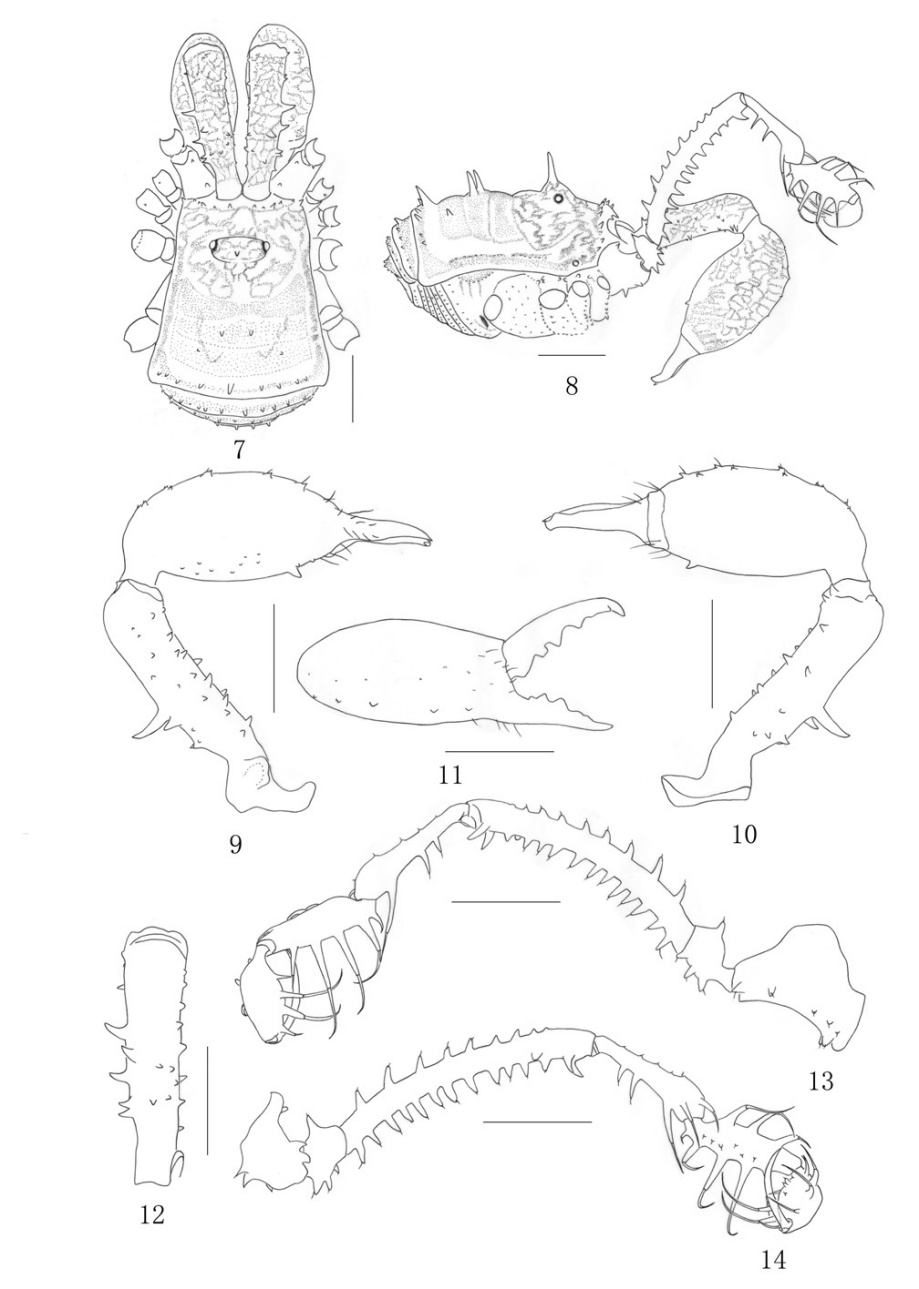
*Plistobunus columnarius* sp. n. male **7** Body, dorsal view **8** Same, lateral view **9** Left chelicera, medial view **10** Same, ectal view **11** Distal segment of the left chelicera, frontal view **12** Proximal cheliceral segment, dorsal view **13** Left pedipalpus, ectal view **14** Same, medial view. Scale bars: 1 mm (7–14).

### Plistobunus columnarius sp. n.

urn:lsid:zoobank.org:act:1E2AB18D-7941-495F-A8C5-1406864D6F79

http://species-id.net/wiki/Plistobunus_columnarius

Figs 7–23 

#### Type material.

Holotype male (Opi.11061601), CHINA: Hainan Province, Tunchang County, Xichang Town [N19°26´, E 110°02´], June 16, 2009, C. Zhang leg. (MHBU), paratype 1♀(Opi.11061602), same data as holotype.

#### Diagnosis.

The new species is similar to the type species *Plistobunus rapax* Pocock, 1903, but can be easily distinguished from the latter by: 1) anterior margin of carapace with a row of six setiferous tubercles on either side; 2) the femur of pedipalpus ventrally with 13 setiferous tubercles in male and seven setiferous tubercles in female; 3) the medial surface of cheliceral proximal segment with a huge protuberance at base.

#### Etymology.

The specific name is derived from the Latin adjectives “columnaris” meaning columnar, refers to shape of the stylus in male penis.

#### Description.

Male (holotype) habitus as in Figs 7–8. Coloration: entire body rusty yellow, with somewhat dark brown to blackish brown patches on the dorsum; median area of carapace with blackish brown reticulations; each side of carapace dark brown; lateral ridges of the scute margined with blackish brown; venter concolorous with dorsum; coxae with dark brown reticulations; free sternites with transverse band of blackish brown; chelicerae rusty yellow, with blackish brown reticulate markings above; pedipalpus dark brown, tibia and tarsus paler, tarsus with dark brown reticulations dorsally; legs brown, trochanters yellow, femur, patella and tibia with blackish brown reticulations, metatarsus and tarsus lighter.

##### Dorsum.

Dorsal scutum trapezoid in shape; widest portion of body at forth scutal area. Carapace with a row of six small setiferous tubercles on each side of front margin. Surface of dorsum almost smooth. Ocularium wide oval, remote from anterior border of scutum, armed with a long erect median spine. Opisthosomal region of scutum with four areas, first area well defined, entire. Second area with two long median spines. Third with a pair of hair-tipped tubercles removed from each other. Fourth with a transverse row of nine tubercles, median tubercle enlarged into a spine. Each lateral margin of the scutum with a longitudinal row of minute hair-tipped granules. Free tergites each with a transverse row of hair-tipped granules spread over its entire width.

##### Venter.

All coxae and genital operculum granulate. Coxae I–III disto-dorsally with two coarse tubercles on anterior and posterior sides respectively. Coxa IV with a reduced one on medio-prolaterally. Coxa I medio-ventrally and prolaterally with transverse rows of hair-tipped tubercles. Coxae II–III medio-ventrally with a transverse row of same tubercles. Coxa III with prolateral and retrolateral rows of small humps. Coxa IV widened, with hair-tipped granules. Free sternites each with a transverse of hair-tipped granules. Tracheal stigma clearly visible.

##### Chelicera (Figs 9–12).

Fairly strong. Proximal segment elongated and with numerous hair-tipped tubercles above; the dorsal surface centrally with six hair-tipped tubercles, of which distal one the largest, three medium-sized tubercles posterior to it, and two smaller ones toward the medial side; the ventral surface and the medial surface with rows of eight hair-tipped tubercles respectively, the medial surface with a huge protuberance at base; the ectal surface with a row of seven tubercles. Second segment considerably widened, medially with five enlarged hair-tipped bifid tubercles and ectally with five reduced ones on prodorsal surface, medially with many small hair-tipped granules on ventral surface, the largest one towards the base of fingers. Fingers relatively strong, inner edges toothed as illustrated (Fig. 11): moveable finger with four teeth, the proximal one square, the middle with two crest teeth, the distal one rectangular; fixed finger with five teeth, the proximal two formed one bifid tooth, the middle with one conical tooth, the distal with two lower than the middle one.

##### Pedipalpus (Figs 13–14)

Coxa dorsally with one proximal and one strong distal bifurcate setiferous tubercles, ventrally with a row of five setiferous tubercles, two enlarged ones additionally at base. Trochanter ventrally with three setiferous tubercles, dorsally with one. Femur elongate, ventrally with 13 setiferous tubercles, dorsally with nine ones of which the distal two inconspicuous, distally on medial side with two setiferous tubercles of which the distal one tipped and the other one conical. Patella very long, widening abruptly distally, with two setiferous tubercle disto-medially and three setiferous tubercles ectally, dorsally with five inconspicuous granules. Tibia with three medial and five ectal setiferous tubercles, with a row of six hair-tipped granules in the ventral surface. Tarsus with four setiferous tubercles on both sides of ventral surface, with three granules in the ventral surface. Tarsal claw nearly as long as tarsus, strongly curved.

##### Legs.

**Figures 15–22.**
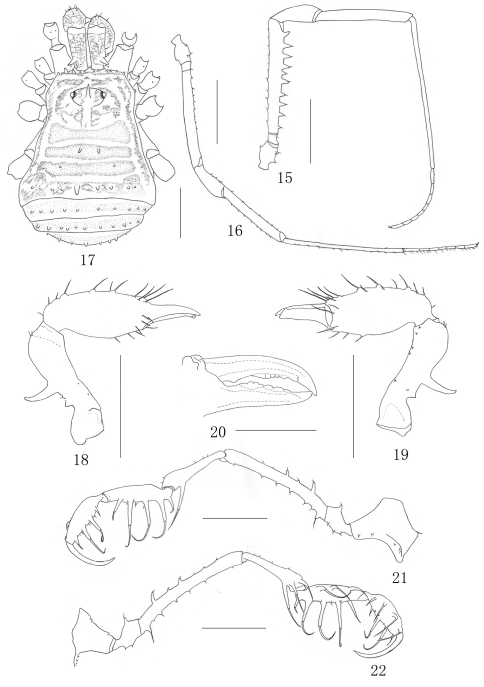
*Plistobunus columnarius* sp. n. **15** Right first leg, male, retrolateral view **16** Left first leg, female, retrolateral view **17** Female body, dorsal view **18** Left chelicera, female, medial view **19** Same, ectal view **20** left cheliceral fingers, female, frontal view **21** Left pedipalpus, ectal view **22** Same, medial view. Scale bars: 1 mm (15–19, 21–22); 0.5 mm (20).

Legs I–II slender and legs III–IV strong. Trochanters I–II each with one hair-tipped tubercles arising distally on the dorsal surface, the ventral surface with three hair-tipped tubercles. Trochanters III-IV smooth dorsally, with inconspicuous granules ventrally. Femur I–II ventrally with a row of 10 or 17 setiferous tubercles respectively (Fig. 15). Femur III ventrally with two rows of 12 and 14 setiferous tubercles respectively. Femur IV ventrally with two rows of many granules. The remaining leg-segments unarmed, smooth, with hairs. Tarsi III–IV with bare double claws, without scopulae. Tarsal formula: 8/17/7/8. Distitarsus of the first and second tarsi with two segments.

##### Penis (Figs 25–31).

slender, its shaft widened distally. The apical structure is divided by lateral incisions into both DP and VP. The VP with complex structures, is separated again by a median cleft, consists of two LPs and the membrane between both sides. The DP simple, distal margin corrugate. G protruding beyond the anterior margin of the dorsal surface. SL somewhat as the shape of tulip ventrally and dorsally, consists of VSL and DSL. The VSL slightly curved toward ventral surface and with a labiate protrusion distally. The DSL petaloid. S smooth, columnar and arising straight from the glans, SL almost entire surrounding the S. BS globular, well developed, immovable and entire sunken into truncus. Setae arranged as follow: 11 VS, four DS, four LS, four GBS.

##### Female (Figs 16–24).

In general appearance similar to the male but smaller and with abdomen more rounded posteriorly (Fig. 17). Chelicera (Figs 18–20) smaller and with reduced tubercles, the proximal segment shorter than those of the male, the second segment is not so greatly enlarged as in the male, inner edges of finger toothed as illustrated (Fig. 20). The pedipalpus (Figs 21–22) femur with seven reduced setiferous tubercles ventrally and two conspicuous setiferous tubercles dorsally, distally on medial side without any setiferous tubercle; Patella with two setiferous tubercle disto-medially and one setiferous tubercles disto-ectally. Setiferous tubercles of leg I (Fig. 16), as well as leg II–IV inconspicuous. Tarsal formula: 7/17/7/8. Distitarsus of the first and second tarsi with two segments.

##### Ovipositor (Figs 23–24).

**Figures 23–31.**
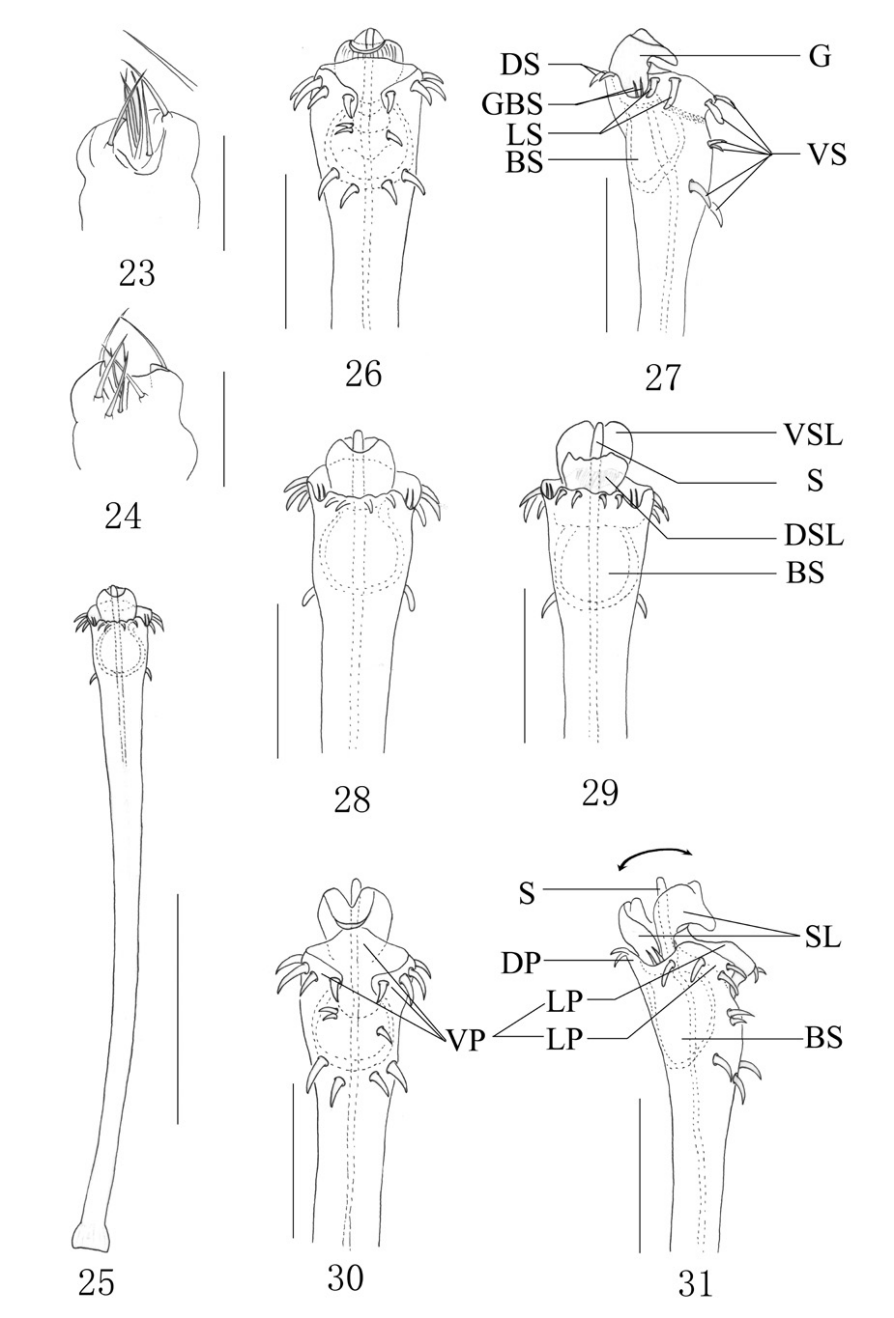
*Plistobunus columnarius* sp. n. **23** Ovipositor, dorsal view **24** Ditto, ventral view **25** Entire penis, dorsal view **26** Penis tip, ventral view **27** Ditto, lateral view **28** Ditto, dorsal view **29** Expanded penis, dorsal view **30** Ditto, ventral view **31** Ditto, lateral view. Abbreviations: **BS** basal sac **DP** dorsal plate **DS** dorsal setae **DSL** dorsal stylar lobe **G** glans **GBS** basal setae of glans **LP** lateral plate **LS** lateral setae **S** stylus **SL** stylar lobe **VP** ventral plate **VS** ventral setae **VSL** ventral stylar lobe. Scale bars: 0.5 mm (25); 0.20 mm (26–31); 0.25 (23–24).

Ventral surface with five setae and dorsal surface with six setae. Tip of each seta pinpoint (Fig. 23).

##### Measurements.

Male holotype (female paratype): body 3.57 (3.37) long, 2.70 (2.65) wide at the widest portion, scutum 3.21 (2.75) long. Ocularium 0.38 (0.33) long, 0.90 (0.68) wide. Pedipalpus claw 0.90 (0.83) long. Penis 1.55 long. Measurements of left pedipalpus and legs as in Table 1.

#### Habitat.

Collected by leaf litter sieving in the rubber forest.

#### Distribution.

China: Hainan (Tunchang County).

#### Remarks.

A certain similarity in ornamentation of the new species may be noted with that of *Plistobunus rapax* as figured and described by (Roewer (1912, 1923, 1938) and Pocock (1903), e. g., the high erect spine on ocularium, fused scutal areas I–II, a pair of spines on the second opisthosomal area, a spine on the fourth opisthosomal area of the dorsal scutum, the greatly elongate pedipalpus and almost identical chelicera. The style of the ornament of the new species is typical of *Plistobunus*. Based on above we believe it should be a new species.

Martens (1988) suggested that “an intensive search, not only for diverse morphological structures in laniatorid penes, but even more for the function of their movable parts under hemolymph pressure will reveal a wealth of structures up to now largely undiscovered”. Many families (e. g., Assamiidae Sørensen, 1884; Biantidae Thorell, 1889; Fissiphalliidae Martens, 1988; Oncopodidae Thorell, 1876; Phalangodidae and Podoctidae Roewer, 1912) were studied in genital morphology and function. At the meantime, some laniatorid structure cannot be homologized completely. Epedanidae is restricted in Asia, among of these known species, Suzuki porvided drawings of genital morphology, however, he failed to give the expanded the structures of the penis.

In this paper, we tentatively explain the movement of the penis in the new species briefly. The S is mainly exposed by the movement of VSL and DSL in the opposite direction, DSL tends to move dorsally wider than that of VSL. DSL and VSL like the petal, S is similar to the stamen. The expansion of G resembles the blooming flower (Fig. 31).

**Table 1.** Pedipalpus and leg measurements of the male holotype (female paratype).

	Trochanter	Femur	Patella	Tibia	Metatarsus	Tarsus	Total
Pedipalpus	0.63(0.50)	2.63(2.00)	1.35(1.13)	1.18(1.00)		0.95(0.88)	6.74(5.51)
Leg I	0.50(0.45)	2.10(1.68)	0.80(0.58)	1.50(1.25)	2.48(1.88)	1.38(1.20)	8.76(7.04)
Leg II	0.50(0.45)	2.98(2.45)	0.83(0.75)	2.35(1.88)	3.13(2.43)	2.60(2.38)	12.39(10.34)
Leg III	0.50(0.45)	2.38(2.00)	0.85(0.63)	1.63(1.38)	2.95(2.45)	1.43(1.25)	9.74(8.16)
Leg IV	0.50(0.45)	3.00(2.70)	0.95(0.75)	2.00(1.75)	3.95(3.25)	1.75(1.45)	12.15(10.35)

## Discussion

Kury (1993) detached the family Epedanidae which included four subfamilies (i. e., Epedaninae, Acrobuninae, Dibuninae and Sarasinicinae) from the Phalangodidae for the first time. Most genera and species were created by Roewer, based on external morphological characters. Suzuki finely described and redescribed some species of Epedanidae, especially for the male penis. However, internal relationships of the four subfamilies have not been investigated (Kury 2007).

It is obvious to assign *Plistobunus* to the Epedanidae because its penis possesses immovable sac, and protruding glans which only consists of stylus and stylar lobe. Furthermore, according to Kury (2007), the genus should belong to the subfamily Epedaninae based on morphological characters such as eyes placed laterally at the base of a well-marked common ocularium, tarsi III–IV without scopula and distitarsus I with two tarsomeres.

The external morphology of *Plistobunus* is similar to the genera *Euepedanus* Roewer, 1915 and *Pseudoepedanus* Suzuki, 1969. *Plistobunus* is distinct from *Euepedanus* and *Pseuduoepedanus* by the number of tubercles on the femur of pedipalpus ventrally and dorsally. The most significant difference concerns the male genitalia. *Euepedanus* has conspicuous ventral plate which is absent in *Plistobunus*; and glans in *Euepedanus* protrude from the center at the top of penis, while glans in *Plistobunus* protrude sideways beyond the distal penis and near the dorsal plate; dorsal plate of penis has incision in *Pseudonepedanus*, while absent incision in *Plistobunus*; and the shape of glans are different in both *Pseudonepedanus* and *Plistobunus*.

The male genitalia of *Plistobunus columnarius* sp. n. is very similar to some other species of Epedaninae, Acrobuninae, Sarasinicinae and even *incertae sedis* of Epedanidae, respectively *Alloepedanus robustus* Suzuki, 1985 (Epedaninae), *Zepedanulus ishikawai* Suzuki, 1971 (Epedaninae), *Zepedanulus watanabei* Suzuki, 1981 (Epedaninae), *Toccolus chibai* Suzuki, 1976 (Epedaninae), *Toccolus globitarsis* Suzuki, 1969 (Epedaninae), *Paracrobunus bimaculatus* Suzuki, 1977 (Acrobuninae), *Opelytus spinichelis* Roewer, 1938 (Sarasinicinae), *Pasohnus bispinosus* Suzuki, 1976 (Sarasinicinae), *Tokunosia tenuipes tenuipes* Suzuki, 1964 (*incertae sedis*), *Tokunosia tenuipes taiwana* Suzuki, 1977 (*incertae sedis*).

Moreover, *Alloepedanus robustus* (Suzuki, 1985: 87–89, fig. 10) which is distributed in the Doi Sutep (Thailand, Chieng Mai Province) has similar external morphology with *Plistobunus columnarius* sp. n. (e.g., ocularium with a long median spine, spination of chelicerae, pedipalpus femur with a row of more than 10 setiferous tubercles dorsally and ventrally, tarsi I–IV with more than six segments, distitarsus I–II with two segments, tarsi III–IV with bare double claws). *Alloepedanus robustus* differs from *Plistobunus columnarius* sp. n. by penis without dorsal plate and the scutal area II without two long median spines.

Similar concerns apply to *Toccolus globitarsis* (Suzuki, 1969: 91–96, figs 9–11), *Toccolus chibai* (Suzuki, 1976: 15–18, fig. 5), *Opelytus spinichelis* (Suzuki, 1976: 18–20, fig. 6) and *Pasohnus bispinosus* (Suzuki, 1976: 21–23, fig. 8), *Toccolus globitarsis* is distributed in the Paktong Chai (Thailand) and others in the Pasoh Forest Reserve (Malaysia). However, *Plistobunus bispinosus* can be easily distinguished from *Plistobunus columnarius* sp. n. by two long median spines on the scutal area III instead of the scutal area II.

The external morphology of other four species (i. e., *Zepedanulus watanabei*, *Zepedanulus ishikawai*, *Tokunosia tenuipes tenuipes*, *Tokunosia tenuipes taiwana* and *Paracrobunus bimaculatus* ) is distinct from *Plistobunus columnarius* sp. n. except *Alloepedanus robustus*, *Toccolus globitarsis*, *Toccolus chibai* and *Pasohnus bispinosus*. They are all absent a long median spine on ocularium and without conspicuous enlarged tubercles on proximal segment of chelicera. They are distributed mainly in the Thailand (e. g., *Zepedanulus watanabei* (Suzuki, 1981: 268–269, fig. 1)), Ryukyus (e. g., *Zepedanulus ishikawai* (Suzuki, 1971: 196–200, figs 22–30), *Tokunosia tenuipes tenuipes* (Suzuki, 1971: 193–196, figs 12–21)), Taiwan Island (e. g., *Tokunosia tenuipes taiwana* (Suzuki, 1977b: 124–125, fig. 1)), Philippines (e. g., *Paracrobunus bima culatus* (Suzuki, 1977a: 17–20, figs 5–6)).

Based on the references cited above, these species are distributed mainly in Southeast Asia. Furthermore, the male genital morphology of most species also shows great similarity to *Plistobunus columnarius* sp. n., although they belong to different subfamilies presently. For these reasons, we presume they may have a relatively close phylogenetic relationship, not depend only on the external morphological characters which currently support the subfamilies.

## Supplementary Material

XML Treatment for Plistobunus

XML Treatment for Plistobunus rapax

XML Treatment for Plistobunus columnarius
